# Management of endocrino-metabolic dysfunctions after allogeneic hematopoietic stem cell transplantation

**DOI:** 10.1186/s13023-014-0162-0

**Published:** 2014-10-29

**Authors:** Marie-Christine Vantyghem, Jérôme Cornillon, Christine Decanter, Frédérique Defrance, Wassila Karrouz, Clara Leroy, Kristell Le Mapihan, Marie-Anne Couturier, Eva De Berranger, Eric Hermet, Natacha Maillard, Ambroise Marcais, Sylvie Francois, Reza Tabrizi, Ibrahim Yakoub-Agha, On behalf of the Société Française de Greffe de Moelle et de Thérapie Cellulaire (SFGM-TC)

**Affiliations:** Lille University Hospital, Endocrinology and Metabolism, Lille, France; INSERM, U859 Diabetes Cell Therapy, Lille, France; Loire Cancerology Institut, Hematology, Saint Priest En Jarez, Lille, France; Lille University Hospital, Endocrine Gynaecology, Lille, France; Morvan Hospital, Hematology, Brest, France; Lille University Hospital, Pediatry, Lille, France; Estaing Hospital, Hematology and Cell Therapy, Clermont-Ferrand, France; Miletrie University Hospital, Hematology, Poitiers, France; Necker Hospital, Hematology, Paris, France; Angers University Hospital, Hematology, Angers, France; Haut Leveque Hospital, Hematology, Pessac, Bordeaux, France; Lille University Hospital, Hematology, Lille, France

**Keywords:** Bone marrow transplantation, Allogeneic hematopoietic stem cell transplantation, Endocrine complications, Gonadal failure, Hypothyroidism, Osteoporosis, Diabetes, Dyslipidemia, Cardiovascular tisk, Secondary cancers, Transplantation de moëlle osseuse, Allogreffe de cellules souches hématopiétiques, Complications endocrines, Insuffisance gonadique, Hypothyroidie, Ostéoporose, Diabète, Dyslipidémie, Risque cardiovasculaire, Cancers secondaires

## Abstract

Allogeneic hematopoietic stem cell transplantation is mainly indicated in bone marrow dysfunction related to blood diseases, but also in some rare diseases (adrenoleucodystrophy, mitochondrial neurogastrointestinal encephalomyopathy or MNGIE…). After decades, this treatment has proven to be efficient at the cost of numerous early and delayed side effects such as infection, graft-versus-host disease, cardiovascular complications and secondary malignancies. These complications are mainly related to the conditioning, which requires a powerful chemotherapy associated to total body irradiation (myelo-ablation) or immunosuppression (non myelo-ablation). Among side effects, the endocrine complications may be classified as 1) hormonal endocrine deficiencies (particularly gonado- and somatotropic) related to delayed consequences of chemo- and above all radiotherapy, with their consequences on growth, puberty, bone and fertility); 2) auto-immune diseases, particularly dysthyroidism; 3) secondary tumors involving either endocrine glands (thyroid carcinoma) or dependent on hormonal status (breast cancer, meningioma), favored by immune dysregulation and radiotherapy; 4) metabolic complications, especially steroid-induced diabetes and dyslipidemia with their increased cardio-vascular risk. These complications are intricate. Moreover, hormone replacement therapy can modulate the cardio-vascular or the tumoral risk of patients, already increased by radiotherapy and chemotherapy, especially steroids and anthracyclins… Therefore, patients and families should be informed of these side effects and of the importance of a long-term follow-up requiring a multidisciplinary approach.

## Résumé

La transplantation allogénique de cellules souches hématopoïétiques est pratiquée dans les dysfonctions de moelle osseuse liées à des hémopathies, mais également dans certaines maladies rares (adrénoleucodystrophie, encéphalomyopathie mitochondriale gastro-intestinale ou MNGIE…). Après quelques décennies, ce traitement a fait la preuve de son efficacité au prix d’un certain nombre d’effets secondaires précoces ou plus tardifs tels qu’infection, réaction greffon-contre-hôte, complications cardiovasculaires et cancers secondaires. Ces complications sont principalement liées au conditionnement, qui requiert une chimothérapie puissante associée à une irradiation corporelle totale (conditionnement myélo-ablatif) ou une immunosuppression (conditionnement non myélo-ablatif). Parmi ces effets secondaires, les complications endocrines peuvent être classées en 1) déficits hormonaux endocrines (particulièrement gonado- and somatotropes) liés aux conséquences de la chimio- et surtout de la radiothérapie, avec ses effets propres sur la croissance, la puberté, l’os et la fertilité; 2) maladies auto-immunes, particulièrement dysthyroidies; 3) tumeurs secondaires impliquant soit les glandes endocrines (cancer thyroidien) ou dépendant du statut hormonal (cancer du sein, méningiome), favorisées par la dysrégulation immunitaire et la radiothérapie; 4) complications métaboliques, spécialement diabètes cortico-induits et dyslipidémies qui s’accompagnent d’un risque cardiovasculaire accru. Ces complications sont intriquées. De plus, l’hormonothérapie substitutive peut moduler le risque cardio-vasculaire et tumoral de ces patients, déjà accru par la radiothérapie et la chimothérapie, notamment les glucocorticoides et les anthracyclines… C’est pourquoi ces personne et leur famille doivent être informées de ces effets secondaires et de l’intérêt d’un suivi multidisciplinaire à long-terme.

## Introduction

Allogeneic hematopoietic stem cell transplantation (allo-HSCT) has become the treatment of choice for a variety of hematological disorders as well as for some rare diseases such as adrenoleucodystrophy and mitochondrial neurogastrointestinal encephalomyopathy (MNGIE). With the increased number of long-term survivors, attention is now focused on the early and late transplant-related complications, which not only can be life-threatening, but also may worsen quality of life of patients [1,2].

In addition to well-known secondary cancer and cardio-metabolic disorders, endocrine dysfunctions have been described in both children and adults in combination with bone complications. Recipient age, underlying disease and transplantation modalities, specifically conditioning regimen (chemotherapy, total body irradiation (TBI)) and graft-versus-host disease (GVHD) prophylaxis (immunosuppression, steroids), influence the occurrence of such complications [3–6]. In gross, endocrine complications may be classified into 1) hormonal endocrine deficiencies (particularly gonado- and somatotropic influencing growth, puberty, bone and fertility); 2) auto-immune diseases, particularly dysthyroidism [7]; 3) secondary tumors, the risk of which is 24 fold higher [8] compared to the general population, involving either endocrine glands (thyroid carcinoma) or dependent on estrogen and progestin (breast cancer, meningioma); 4) metabolic complications, especially steroid-induced diabetes, associated to increased cardio-vascular risk. This last group of complications is less specific and may be encountered in all types of transplantation, even if the cardiovascular risk seems worserned by TBI. Moreover, hormone replacement therapy can modulate the cardio-vascular or the tumoral risk of patients.

Five to 10% of patients die of non-relapse causes at a median of 5 years post allo-HSCT (mainly chronic GVHD, secondary malignancy and infection) [9]. The late endocrine and metabolic complications are hypogonadism in one third of patients, osteopenia/osteoporosis and hypertension in 20 to 25%, and hypothyroidism, dyslipidemia, diabetes and sexual dysfunction in 5 to 10% of 10-year survivors [9]. A careful monitoring of these intricate complications is warranted and requires a multidisciplinary approach.

The main aim of this article is to give an overview on transplant-related endocrine dysfunctions. Our methodological approach consisted in a PubMed review using the following key-words: hematopoietic stem cell transplantation and hypogonadism, thyroid, pituitary, adrenal, bone, iron overload, lipodystrophy, diabetes, dyslipidemia and cardiovascular risk. A multidisciplinary discussion was held based on this literature review and the clinical experience of each author.

## Endocrine complications

### Post-transplant gonadal failure

Central or peripheral gonadal failures (i.e. premature ovarian and testicular failure) are frequent complications of allo-HSCT [10]. Gonadal failure can lead to sex-steroid deficiencies, resulting in infertility. Furthermore, irradiation of the uterus favors poor embryo implantation related to significant alteration of the vascular bed. In the event of pregnancy, the risk of intra-uterine growth retardation, prematurity and uterine rupture is increased. Lastly, children having received an allo-HSCT can present failure to thrive and delayed puberty.

#### Main factors of gonadal failure

Gonadal failure mainly depends on the conditioning treatment. High-dose busulfan, TBI and testicular irradiation are responsible for gonadal failure. Although most of the literature series are modest in size, the frequency of delayed puberty is evaluated to be 10% after exposure to cyclophosphamide, 35% after cyclophospamide-busulfan combination therapy, 65% after (10 gray) TBI, and 80% if testicular irradiation is also required.

Alkylating agents such as procarbazine and cyclophosphamide induce prolonged azoospermia in over 90% of men and premature ovarian failure in 5%-25% of women under the age of 30. The risk of infertility increases with a younger age of treatment and the cumulative dose of alkylants. Doxorubicin-bleomycin-vinblastine-dacarbazine chemotherapy is associated with a limited risk of premature ovarian failure and infertility (below 10%).

Overall, large epidemiological studies have revealed the risk of infertility to be at least 40% at age 35 regardless of the age at the time of chemotherapy and even after full recovery of the initial follicular reserve [10,11].

#### Diagnosis

On the biochemical level, hormone deficiency favors dyslipidaemia and insulin resistance. Blood testosterone and oestradiol levels are abnormally low and plasma gonadotropin levels (LH and FSH) are high. Gonadotropin levels may be intermediate or low in cases of additional gonadotropin deficiency. It is essential to rule out other causes of amenorrhea, such as hypothalamic amenorrhea or polycystic ovary syndrome. Low anti-Müllerian hormone (AMH) blood levels reflect a low ovarian reserve and provide information on fertility [12].

#### Preservation of fertility

Cryopreservation of sperm must be considered prior to cytotoxic treatment (i.e. the chemotherapy and/or irradiation) in post-pubertal boys. As soon as the disease has been diagnosed, a female patient must be informed about infertility risk associated with cytotoxic treatments and about fertility preservation techniques that can be applied in her particular situation [13–16] (Table 1).

**Table 1:** **Main techniques of fertility preservation**

**Techniques**	
Ovarian cortex freezing	- for future orthotopic graft or
	- *in vitro* follicular maturation if a graft is not possible because of the risk of re-introducing dormant cells
	**- can even be performed in an emergency**
Oocyte cryopreservation after a conventional *in vitro* fertilization stimulation protocol	- specially indicated in young, single female patients (live birth rate: 1% to 2%)
	- but time for stimulation needed before radio/chemotherapy
*In vitro* fertilization and embryo freezing	- currently the best preservation technique
	- requires *3 weeks* for the ovarian stimulation and thus will only be indicated if it is possible to postpone radio/chemotherapy.
Drug-based preservation with gonadotropin releasing hormone (GnRH) agonists.	- widely used
	- easy to perform, even in an emergencuy context
Transposition of the ovaries prior to radiotherapy.	If the planned treatment is highly gonadotoxic, it is possible (and indeed advisable) to transpose one ovary and freeze the other.
**Indications**	
Young girl	ovarian freezing only possible option
Pubertal girl and /or woman under the age of 40:	all of the above-listed techniques can be considered.
Single women,	oocyte cryopreservation and ovarian cortex freezing preferable

#### Induction of amenorrhea in female transplant recipients

In menstruating women, the induction of amenorrhea usefully decreases the risk of hemorrhage when the platelet counts falls. The most current approach involves administration of a GnRH agonist, which also provides a protective effect on ovarian reserve (Table 2).

**Table 2:** **Induction of amenorrhea**

1.	GnRH agonist (3.75 mg/month subcutaneous leuprolide acetate, in general), enabling
	**a**. induction of amenorrhea
	**b**. “parenteral” contraceptive effect effective even in patients with vomiting
	**c**. possible anti-apoptotic, protective effect on the follicles, observed in animal studies but not yet in women.
2.	In order to avoid flare-ups, it is recommended to prescribe
	**a**. a contraceptive pill 10 to 15 days after the agonist injection
	**b**. or a macroprogestin for the first month.

### Corticotropin deficiency following corticosteroid therapy

#### Main factors of corticotropin deficiency

The prolonged use of glucocorticoids can lead to corticotropin deficiency via inhibition of the production of hypothalamic corticotrophin releasing hormone (CRH) and pituitary ACTH. Generally, this corticotropin deficiency resolves spontaneously once steroid therapy is withdrawn, except when the treatment is prolonged or involves high doses. Corticotropin deficiency can also be caused by TBI; this often develops over several years and may be accompanied by other pituitary hormone deficiencies [17]. Last but not least, numerous drugs inhibiting the cytochrome p450, such as macrolides or antifungal drugs, can interfere with steroid metabolism, especially fluticasone and budesonide metabolism, inducing a clinical pseudo-Cushing syndrome contrasting with a biological profile of corticoptropin deficiency.

#### Diagnosis

The symptoms of chronic adrenal dysfunction in bone marrow transplant recipients can mimic graft-versus-host reaction, inasmuch as they include fatigue, weakness, anorexia, nausea, vomiting, weight loss, and orthostatic hypotension. The clinical diagnosis of these insidious forms is often problematic and the physician should not hesitate to run laboratory tests, especially morning blood cortisol.

**Figure 1:**
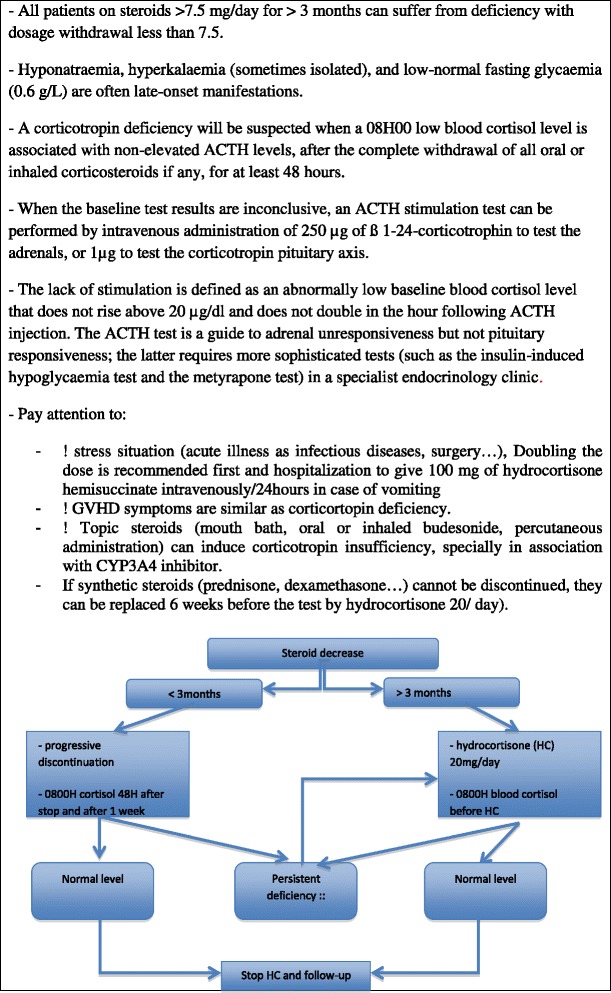
**Corticotropin deficiency.**

The steps of biological diagnosis are given in Figure 1.

#### Treatment

When a corticotropin deficiency has been confirmed by laboratory test results, it is advisable to replace synthetic corticosteroids by 20 mg/day of natural hydrocortisone, if the intention was to stop synthetic cortico-steroids, and to introduce 20 mg/day of hydrocortisone in two separate doses if the patient was not treated (corticotropin deficiency post-radiotherapy). In fact, hydrocortisone has a much shorter half-life and less suppressive activity than synthetic glucocorticoid and may enable the recovery of normal corticotropin function after just a few weeks or as much as one year [18] (Figure 1). Otherwise, the patient should be advised to increase his or her steroid dose in case of intercurrent event (infection, surgery or even hot weather…).

### Post-transplant thyroid disorders

#### Disorders of thyroid function

Besides TBI and immunosuppressive drugs (tyrosine kinase inhibitors, bexarotene, alemtuzumab, interferon alpha, ipili- and iremeli-mumab, thali- and enali-domide) [19]), amiodarone, radiological contrast medium, iodine antiseptic solutions might participate in thyroid dysfunction by massively increasing iodine supply.

*Hypothyroidism* Primary hypothyroidism occurs in about 50% of irradiated patients in the year following radiotherapy. The incidence is proportional to the dose received and the youngest age of treatment. Hypothyroidism may be compensated or transient and the long-term incidence is about 20% [20–22]. Although peripheral hypothyroidism is the most frequent manifestation, thyrotropin deficiency (after bexarotene treatment), and anomalies of the peripheral metabolism of thyroid hormones may also be encountered.

Given the difficulty of diagnosing hypothyroidism in patients whose complaints (asthenia, cold intolerance, etc.) can easily be attributed to the causal disease or chemotherapy itself, it is important to regularly monitor not only plasma thyroid-stimulating hormone (TSH), but also free T4 values. Moreover, taking into account the sometimes fluctuating values of TSH in these patients, it is important to keep in mind that isolated, mild increase of TSH below 10 mUI/L should always be confirmed a second time before making a treatment decision, according to the recommendations of hypothyroidism treatment [23,24]. Otherwise, the administration of iron per os must not be given at the same time as replacement hormone therapy with L-thyroxine since it alters the absorption of the hormone by interfering with the entero-hepatic cycle of bile acids.

*Hyperthyroidism* The donor-recipient transfer of B and T lymphocytes may favor the occurrence of hyperthyroidism (Graves disease, sometimes preceded by a hypothyroid episode related to blocking anti-TSH receptor antibodies or immune reconstitution syndrome). However, the disappearance of an autoimmune disease has also been reported [25]. Iodine overload can also promote hyperthyroidism and stress may trigger the appearance of Basedow’s disease.

#### Thyroid nodular dystrophy

The 10-year cumulative incidence of thyroid nodules in ultrasound sonography in children after TBI is estimated at 16%, half of them potentially malignant. Ten-year post-HSCT is the median time of occurrence [22].

#### Thyroid cancer

In a large retrospective European study of allo-HSCT including about 69,000 cases, the relative risk of thyroid cancer was three-fold higher than in the general population. A multivariate analysis showed that the younger the patient’s age at transplantation, the greater the risk (with relative risks greater than 20 before the age of 10 and nearly 5 between the ages of 11 and 20). Irradiation, female gender and the presence of a GVHD were also identified as risk factors. A third of the patients were asymptomatic when thyroid cancer was discovered and all but one of the 32 patients responded to standard treatment (thyroidectomy and iodine-131 therapy) [26]. Therefore, this higher incidence of thyroid cancer was not associated with a worse outcome after allo-HCST, as also demonstrated after transplantation even if the initial presentation was more aggressive [27,28].

### Pituitary deficiencies following radiotherapy

TBI can trigger late-onset anterior pituitary deficiency occurring 2 to 10 years after treatment and slowly worsening over time. The onset of these corticotropin (see above), gonadotropin, thyreotropin, somatotropin deficiencies is very insidious and is often revealed by non-specific asthenia, paleness, hair thinning, a loss of libido and (in menstruating women) oligo- or a-menorrhoea. On the biochemical level, pituitary as well as thyroid, adrenal or sex hormone levels do not drop sharply but tend to fluctuate around low-normal values. Dynamic tests in a specialized endocrinology clinic are often required for confirmation of the diagnosis and adjustment of the treatment.

#### Corticotropin deficiency

Besides post-steroid corticotropin deficiency, TBI can also induce pituitary damage, with an ACTH deficiency, which will be confirmed either with a low dose corticotropin test or with a hypoglycaemic or a metyrapone test performed in a specialized clinic. The treatment with hydrocortisone may unveil thyrotropin deficiency, which should always be checked after hydrocortisone replacement therapy.

#### Thyroid dysfunction

The blood TSH level is below 5 mIU/L and free T3 and free T4 levels are low-normal or below normal. The treatment must be initiated progressively – especially when the person is elderly or has underlying coronary disease and when the deficiency has been present for some time. This is done to reach free thyroid hormone levels within the upper quartile of the normal range. In fact, a TSH assay is of little value to adapt treatment in central hypothyroidism.

#### Somatotropin deficiency

Young age at irradiation and the absence of dose fractionation in TBI are predictive of growth hormone (GH) deficiency and growth retardation in 20-50% of the patients having received brain irradition or TBI in addition to chemotherapy. This deficiency results in small adult height stature, which may also be favored by skeletal anomalies and gonadal failure [29,30].

An undetectable GH concentration and low plasma IGF1 concentration, which does not exceed 3 ng/ml after stimulation, are suggestive of somatotropin deficiency. If treatment is considered (particularly in children with growth retardation), it is necessary to perform two dynamic tests in a specialist clinic. It is important to note that GH therapy has been shown to help gain +1.1 SD of height after 5 years, whatever the GH secretion status was before treatment [31]. Nevertheless, as this treatment type can only be administered after the initial disease has been controlled, the indication of the treatment should be carefully weighed. In adulthood, a severe alteration in overall health status due to somatotropin deficiency may prompt consideration of GH treatment. Given that the literature’s data are contradictory about the risk of cancer recurrence, it appears wise to only suggest this treatment when the clinical impact is severe and when close monitoring is possible.

#### Gonadotropin deficiency

As described above, low oestrogen or testosterone levels and non-elevated gonadotropins levels are suggestive of gonadotropin deficiency that is amenable to hormone replacement therapy [29,30].

### Other endocrine dysfunctions

#### Parathyroid disorders

Adenomatous hyperparathyroidism is a rare but well-known complication of radiotherapy [32] and should be considered after the discovery of osteopenia or kidney stones. Referral for thyroid disease is another frequent circumstance of diagnosis (10% of these patients also have a parathyroid adenoma) [33]. Besides the mutagen effect of TBI, a chronic stimulation of PTH in response to the irradiation-altered bone marrow microenvironment, could also favor hyperparathyroidism, often all the more so when kidney function is altered [34].

#### Osteoporosis

Gonadal failure, corticosteroid therapy, the lack of physical activity engendered by asthenia, denutrition and vitamin D deficiency are all risk factors for osteoporosis in light of advice to avoid sun exposure is given to prevent skin cancer in cases of TBI [35].

In a French prospective multicentric cohort of childhood leukemia survivors, transplantation with gonadal insufficiency and female gender where the 2 main factors associated with lower femoral bone mineral density, whereas the adult patients had a slight reduction in lumbar bone mineral density, whatever their transplantation status [36]. Concerning chimiotherapy, only high-doses cause osteoporosis with respectively a 10% and 20% loss in cortical and trabecular bone by altering bone progenitors [37]. This condition can be worsened by somatotropin deficiency, hyperthyroidism or additional hyperparathyroidism. It is thus essential to prevent osteoporosis – and perhaps GVHD [38], by providing sufficient vitamin D supplementation (100,000-unit dose of oral cholecalciferol monthly, together with 500 mg to 1 g of calcium per day or a sufficient intake of dairy products (two yogurts and 30 g of cheese providing about 600 mg of calcium/day). The treatment of gonadal insufficiency is essential [39]. Screening with dual-energy X-ray absorptiometry is useful when several risk factors are present. Levels of bone remodeling markers (osteocalcin and procollagen type I N-terminal propeptide for bone formation and carboxy-terminal telopeptide of type I collagen or CTX for resorption) do not change significantly [40].

#### Lipodystrophies and metabolic syndrome

GVHD sometimes prompts the appearance of localized lipodystrophies – particularly in subcutaneous abdominal tissue, which takes on an infiltrated, cellulite-like, orange-peel aspect. Given that adipose tissue is a key player in innate immunity and the regulation of insulin resistance, it is possible that these acquired lipodystrophies result from the immune deregulation induced by transplantation and are involved in the pathogenesis of metabolic syndrome [41]. mTOR inhibitors block the maturation of adipocytes, which may mean that the latter drug will be of particular value for the treatment of this type of lipodystrophic GVHD.

#### Iron overload

Outside of the specific problem of ß thalassemia, hyperferritinemia is found in 93% of children one year after allo-HSCT but diminishes with time. Hyperferritinemia is correlated with gamma glutamyl-transferase levels and is a well-known factor of liver insulin resistance, but also insulinopenia and therefore diabetes. Iron overload, better assessed by liver MRI than by blood ferritin level, is probably involved in endocrine dysfunction, since it is significantly greater in patients suffering from hypothyroidism or a somatotropin deficiency. Nevertheless its influence on overall mortality has been diversely appreciated according to the tool used to assess it (ferritin or MRI), the time of assessment (pre- or post-allo-HSCT) and finally the period considered for outcome. No association was found between pretransplant MRI-assessed iron overload and 1-year allo-HSCT outcomes [42], whereas post-HSCT hyperferritinemia or MRI-iron overload has been shown to have a detrimental effect on 5-year outcomes [43,44]. Therefore, this condition may necessitate treatment (usually careful bloodletting, or sometimes iron chelators) when it is symptomatic, persistent, and confirmed by liver-MRI.

Anomalies of iron metabolism also influence FGF23 and phosphorus-calcium metabolism. Indeed intravenous iron administration may induce transient hypophosphatemia [45].

## Metabolic complications

### Post-transplant diabetes

Many transplanted patients develop diabetes and or glucose intolerance despite the absence of being overweight or a family history of diabetes. The cumulative incidence of diabetes plus glucose intolerance was recently estimated at 11% at 5 years and 69% 10 years after allo-HSCT. A higher preprandial glucose level in the peri-HSCT and an age ≥6 year-old at the time of HSCT are predictive factors of glucose tolerance disorders [46]. High doses of steroids, increase insulin resistance and certain immunosuppressive agents used to treat GVHD (such as calcineurin and mTOR inhibitors) inhibit insulin secretion and/or modulate insulin resistance [47]. Furthermore, 80% of patients having received radiotherapy display insulin resistance and 60% dyslipidemia. An increase of the body’s fat mass (especially the visceral adipose tissue), possibly promoted by a GH deficiency, is involved in the pathogenesis of metabolic syndrome in association with high leptin and low adiponectin levels [48,49]. Indeed, the occurrence of post-HSCT diabetes is also related to altered immune regulation, especially of Tregs [50] and could be associated with more frequent GVHD [51].

The term “metabolic syndrome” defines a clustering of cardiovascular risk factors, defined by the National Cholesterol Education Program Adult Treatment Panel III, as the simultaneous occurrence of at least three of the following: abdominal obesity, arterial hypertension, hyperglycemia, hypertriglyceridemia and low high-density lipoprotein cholesterol (HDL-C) (Table 3). Its prevalence is increased after allo-HSCT and its main risk factors are high insulin and leptin levels, older age and hypogonadism [52]. Metabolic syndrome is also favored by stress, lack of physical activity, enteral nutrition, which sometimes leads to liver steatosis [53], gonadal hormone deficiency and (when repeated transfusions are necessary) iron overload.

**Table 3:** **Metabolic syndrome and cardiovascular risk factors**

**Metabolic syndrome**	**Cardiovascular risk factors**
- **insulin resistance**	- **family history of early-onset coronary heart disease:** myocardial infarction or sudden death of the father or a first-degree male relative before the age of 55 or of the mother or a first-degree female relative before the age of 65 or stroke in a family member before the age of 45.
- **pro-thrombotic, inflammatory state**	- **on-going tobacco use** or cessation within the last three years.
- **high blood pressure**	- **hypertension** (even when treated).
- **changes in the distribution of adipose tissue**	-** HDL-cholesterol <0.4 g/L** (NB: values >0.60 g/L are protective).
- **dyslipidemia** characterized by hypertriglyceridemia (above 1.5 g/L) and low HDL cholesterol levels (<0.4 g/L in male and 0.5 g/L in female). leading to early-onset cardiovascular disease.	- **Microalbuminuria >30 mg/24 h.**
	- **Age >50 in men, >60 in women** + history of transplantation
***NB: Role of immunosuppressive regimen***	
- *Glucocorticoid therapy* increases total cholesterol, VLDL, the size and density of LDL particles and TG by increasing insulin resistance.	
- *Calcineurin inhibitors* reduce clearance of athero- genic lipoproteins by increasing the activity of hepatic lipase and decreasing lipoprotein lipase.	
- *Ciclosporine*, metabolized through CYP3A4, induces a greater adverse impact on lipid profiles than tacrolimus, and may increase systemic statin exposure	
- *Sirolimus* causes dyslipidaemia in 50% of cases	

Consultation with a diabetologist is notably required in patients who were diabetic before transplant and those who present poorly controlled steroid-induced diabetes. In this context, the goal of diabetes treatment is to maintain glycaemia between 0.80 and 1.50 g/L and thus minimize polyuric syndrome, water balance/electrolyte disorders, denutrition, infection and the risk of hypoglycemia.

The criteria diagnosis of diabetes and the blood glucose monitoring, dietary, oral anti-diabetic and insulin treatment guidelines are detailed in Tables 4, 5, 6 and 7, in order to enable a non-specialist to provide sufficient care in a patient who is given steroids and/or who reveals post-transplant diabetes. The adjustment of steroid dose and/or immunosuppressive regimen might sometimes resolve the hyperglycaemic state. Diet should never unduly be hypocaloric even if restricted in saturated fat or high glycaemic index food. Otherwise, to start insulin therapy does not mean it will be definitive. Finally, post-prandial blood glucose levels are the first to increase with steroids and their monitoring should not be skipped even if fasting blood glucose is normal.

**Table 4:** **Diagnosis of diabetes and blood glucose monitoring guidelines**

**Diagnosis of diabetes**	- glycaemia ≥2 g/L, associated with clinical signs
	- fasting blood glucose ≥1.26 g/L on two occasions
	- blood glucose ≥2 g/L 2 hours after OGTT
**HbA1c >6%**	- should trigger more frequent blood glucose monitoring,
	- given that the anemia frequently observed in transplant patients
	can alter HbA1c
**In patients with weight loss, thirst and polyuria (particularly at night),**	- capillary blood glucose monitoring must be performed
	- before a meal and two hours thereafter
	- with a ketone research.
**If 2 capillary blood glucose values >1.50 g/L,**	- monitoring must be continued
	- regardless of whether or not the patient is symptomatic.
**When corticosteroid therapy is initiated**	- check postprandial blood glucose ++
	- may be elevated even when pre-prandial glycaemia is
	normal regardless of the clinical signs.
**During a steroid therapy step-down phase**	- frequent monitoring recommended, to avoid hypoglycemia.
**During the period of insulin adjustment**	- capillary glycaemia should be monitored
	- 6 x/day (before each main meal and 2 hours thereafter).
**If not possible to obtain regular self-monitoring**	- try to obtain 6 or 7 measurements over 2 or 3 days
	- or refer the patient for a 3- to 7-days continuous
	ambulatory glucose monitoring
**When nocturnal enteral nutrition is initiated,**	- perform 1 or 2 night-time and a morning capillary blood glucose to adjust the evening dose of insulin.

*Once the treatment parameters have stabilized, monitoring can be relaxed, with measurement of pre-prandial and postprandial glycaemia (at 2 hours) after one of the day’s meals*.

**Table 5:** **Dietary principles in case of diabetes**

**A consultation with a dietician is needed** so that patient learns to: 1*.* identify low glycaemic index food, 2. limit the intake of high glycaemic index food especially between meals	**High-calorie, low glycaemic index foods** should be favoured (oleaginous foods, rice pudding, complex carbohydrate-lipid mixtures etc.…).	**Insulin therapy and self-monitoring** must be adapted to suit the patient’s dietary habits (small, frequent meals rather than few, large meals a day and gastrointestinal GVHD modulating digestive tolerance	**If weight loss, food supplements** *for diabetics can be prescribed.*

**Table 6:** **Guidelines for treatment of post-transplant diabetes**

**Oral antidiabetic agents** have not been studied in terms of efficacy and safety in transplant recipients, pediatric recipients in particular.		**Insulin therapy** is preferred in all unstable situations because of its anabolic effect. The objective is to tailor the insulin therapy to food habits, in order to limit weight loss
Most antidiabetic agents contraindicated in cases of kidney failure and cholestasis.	In contrast, **acarbose** despite digestive side effects and **glinides** can be useful.	- **slow-acting insulin** (often an insulin analogue like detemir (12 hours) or glargine (24 hours))
**- Metformin:** risk of lactic acidosis.	***Repaglinide*** (0.5 mg to 4 mg before each snack) can be used instead of injections of ultra-rapid insulin (particularly in patients on low doses of steroid).	- **rapid-acting insulin** (lispro, aspart or glulisine) (2 hours) or regular insulin (4 hours) administered at meal times.
- **Sulphamides** with a long half-life (such as gliclazide) increase the risk of hypoglycemia.		- NB: ultra-rapid insulin (lispro, aspart or glulisine) can be administered immediately after the end of the meal, when the food intake is somewhat unpredictable
		**NB**
**- Gliptins** sometimes lead to pancreatitis		- Change regularly insulin injection site, to avoid lipodystrophy.
**- GLP-1 agonists** promote nausea and weight loss.		- Adapt length of the needle: 4–5 mm if body weight <40 kg, 6 in a lean, 8 in a normal-weight and 12 mm in an obese person.
		- The patient must learn to recognize and treat symptoms of hypoglycemia:

**Table 7:** **Adjustment of insulin therapy**

**Rapid-acting insulin**			**Long-lasting basal insulin**
3 methods for adjusting rapid-acting insulin			
***Retrospective*** **(** ***sliding scale*** **)** - the simplest- given dose according to pre-prandial blood glucose	***Anticipatory***	***Functional***	Only when pre-prandial glucose >1.20 g/L or when the patient loses weight
	*-* insulin dose according to postprandial glycaemia observed at the same time on preceding days.	***-*** *insulin dose* based on counting carbohydrates - complex	- usually 2 detemir injections
			- or a single glargine injection in the evening at a total dose of 0.3 to 0.5 U/kg/day.
	- more complex to use but	- poorly suited to transient insulin therapy	- Any increase in pre-prandial glycaemia (particularly in the morning) >1.50 g/L necessitates an increase of 2 to 4 units in the dose of slow-acting insulin relative to the previous day.
	- more appropriate if steroids use		

### Hyperlipidemia

In a retrospective analysis of around 350 allo-HSCT recipients, half had a blood cholesterol value over 2 g/L three months post-transplantation. The median cholesterol and triglyceride levels had increased by one third and two thirds respectively in adults, and by 50% and 100% respectively in children relative to pre-transplantation values. Severe hypertriglyceridemia was observed in about 5% of adults and 8% of children.

Dyslipidemia is associated with poor survival in organ transplant recipients. Even after the discontinuation of immunosuppression and adjustments for age, gender and obesity, allo-HSCT recipients appear to be at a higher risk for diabetes and hypertension than their siblings. An early onset cardiovascular disease can occur, which is driven directly by TBI-related atherosclerosis and chemotherapy-linked cardiomyopathy, especially with anthracyclins, and driven indirectly by post-transplant metabolic syndrome.

### Cardiovascular risk

Indeed, some recent clinical cases have shown the occurrence of early, fatal coronary heart disease and congestive heart failure, at a median age of 35, with a median time of death 7.5 years after transplantation [54].

In a retrospective cohort study, transplant recipients with a survival of at least 2 years experienced increased cardiovascular death and cumulative incidence of ischemic heart disease, heart failure, stroke, vascular diseases, and rhythm disorders. These patients also had a higher prevalence of risk factors such as hypertension, renal disease, dyslipidemia, and diabetes, without any difference according to TBI or allogeneic versus autologous graft [55]. This is confirmed by at least 2 other sudies with an especially high risk (15%) in patients with multiple cardiovascular risk factors and pre-HSCT exposure to anthracyclins or chest radiation [56], and in males during the second and third decade [57].

This higher risk should prompt the physician to closely monitor cardiovascular factors in these patients, particularly over the long-term, once the initial disease phase has been controlled, but in patients who can be as young as in their third decade.

No epidemiological and treatment guidelines being available after allo-HSCT, the cardiovascular risk of these patients has been arbitrarily considered to be the same as in a diabetic patient. Hence, LDL-cholesterol must thus be below <1.3 g/L in all transplant recipients and <1 g/L (or even <0.7 g/L) in secondary prevention (i.e. in patients having presented cardiovascular complications) and in primary prevention (i.e. in event-free patients) in high-risk patients defined by: 1) family history of early-onset vascular disease; 2) albuminuria >0.3 g/day or kidney failure; 3) type 2 diabetes for the previous 10 years or more; 4) presenting two cardiovascular risk factors [58].

Dietary and life style guidance as well as the different available drugs and indications are summarized in Tables 8 and 9. A few randomized trials and one systematic review have shown a reduction of cardiovascular morbi-mortality with statins in heart or renal transplant patients [59,60].

**Table 8:** **Hypolipidaemic diet, lifestyle and drugs**

**Dietary and life style guidance**	**HMG-CoA reductase inhibitors (statins)**	**Fibrates**
- decrease the intake of saturated animal fat (e.g. meats, cheese, sauces and fried foods).	- lower blood LDL-cholesterol levels by competitive inhibition of HMG coenzyme A decreasing liver synthesis of cholesterol	- reduce triglyceride levels by 20-50%.
- favour omega-3 fatty acids (flaxseed, canola and walnut oil, wheat germ, soya, mackerel, herring, salmon…).	- improve survival rates in adults with variable cholesterol levels (regardless of whether or not they have a history of coronary heart disease)	- side effects:
- maintain a normal body weight and do adapted regular physical exercise	- probably also beneficial in bone marrow recipients	o gallstones, transit and muscle disorders.
	- efficacy of rosuvastatin > atorvastatin (with the longest half-life) > simvastatin > pravastatin and fluvastatin (which are less expensive).	o Risk increased in combination with a statin or with altered kidney function and with ciclosporine.
	- statins other than fluva-, prava- and rosuvastatin, are metabolized by cytochrome P450 (or CYP3A4)	- Fenofibrate preferred to gemfibrozil because of fewer side effects, although it can sometimes increase creatinine levels.
	- can thus interfere with many drugs*, calcineurin and mTOR inhibitors, methotrexate, cimetidine, grapefruit juice.	
	- CYP3A4 inhibitors should be avoided in combination with calcineurin inhibitors and statins	
	- Statins have liver, muscle toxicity: high-dose (>80 mg) statins must not be prescribed.	

*Main CYP3A4 inhibitors: calcium-blockers (diltiazem, verapamil), macrolides (erythromycin, clarithromycin), azole antifungals (itraco- and keto-conazole), antivirals (rito-, indi-, nelfi- and ampe-navir).

**Table 9:** **Screening of metabolic complications and management of dyslipidemia (see Tables** 5 **and** 6 **for diabetes management)**

**1. Pre, and 3, 6, 12 months post bone marrow transplantation then yearly**	- Triglycerides (TG)
	- Cholesterol: HDL/LDL
	- Fasting blood glucose, HbA1c
**2. Prior to treatment, rule out hypothyroidism, nephrotic syndrome and cholestasis, by checking**	- TSH, free T4
	- 24-hour proteinuria,
	- bilirubin and alkaline phosphatases
**3. Then treat according to the cardiovascular risk (factors listed in Table** 9 **),**	**- If high cardiovascular risk**, dyslipidaemia must be treated.
	- **If low cardiovascular risk**, treat according to
	- severity of dyslipidemia
	- prognosis of the transplantation.
	- liver enzyme profile.
**4. Adjust treatment according to LDL cholesterol and triglycerides levels**	- Dietary and lifestyle measures always advisable.
	- Modify the dose of immunosuppressantif possible as the 1^st^ step
	- If LDL cholesterol high and triglycerides <2 g/L: statin at the lowest dose to limit toxicity. NB pravastatin and fluvastatin, metabolized through alternative pathways = best choice in patients requiring co-administration of cytochrome P3A4 inhibitors
**5. If mixed hyperlipidaemia or resistance to statin, add**	- ezetimibe to statins, rather than increase the statin dose.
	- fibrates but increases the risk of muscle disorders
**6. If isolated hypertriglyceridemia >8 g/L**	- fibrates alone to limit the risk of acute pancreatitis.
**7. Follow-up**	- Liver biology after 15 days and monthly after.
	- If transaminases levels is >5 N and/or muscular pain, treatment must be stopped and CPK evaluation is required.
	- Inform the patient of potential side effects so that he/she can alert his/her primary care physician

Finally, transplant recipients have an elevated cardiovascular risk, the management of which must be adapted to suit the likely survival after treatment of an endocrine disorder. Overall care management must be based on the following five measures: 1) A balanced diet, exercise and maintenance of a normal weight; 2) smoking cessation; 3) HbA1c <6.5%; 4) blood pressure <130/80 and 5) LDL-cholesterol <1.3 g/L or 1.0 g/L, depending on the cardiovascular risk.

## Conclusion

Coexistence of multiple late sequelae is common in HSCT survivors related to young age at the time of HSCT, high radiation dose, and history of chronic GVHD. The main endocrino-metabolic complications are hypogonadism and infertility on the one hand and diabetes with dyslipidemia and an increased cardiovascular risk on the other hand. These conditions must be screened for and treated (Tables 9 and 10). On-going monitoring must take account of the increased risk of secondary cancer (notably breast cancer, with an incidence of 30% thirty years after radiotherapy). It is therefore essential to maintain long-term follow-up for patients in whom the initial disease has cured.

**Table 10:** **Screening of the most frequent endocrine complications after allo-HSCT**

**Thyroid Disorder**	- TSH, free serum T4 at 6 months and yearly
	- Clinical thyroid examination yearly
	- Sonogram if clinical anomaly
	- If abnormalities are detected, consider referral to an endocrinologist
**Gonad dysfunction/Fertility**	**1. before allo-HSCT:** conservation must be proposed as possible:
	- Man: Sperm collection. After chemotherapy, it is possible if patient is not azoospermic.
	- Female: ovary or oocyte freezing ; ovarian blocking by Gn-RH analogs
	- Prepubertal: freezing of testicle pulp and ovarian tissu sample.
	**2. after allo-HSCT (first months):** contraception is necessary (see Table 2)
	**3. after allo-SCT (second period):**
	- Woman: hormonal assessment and substitution indicated in 6–12 months. Gynecologic evaluation yearly. Be careful between vaginal GVHD and menopausal symptoms.
	- For male, dosage of testosterone if symptoms warrant and consider referral to specialist.
	**4. If pregnancy is discussed:** 2 years between allo-HSCT and pregnancy is the minimum required. Patient should be referred to specialist in assisted reproductive technologies/oncofertility.
**Osteoporosis**	- Compensate a potential deficiency of calcium and vitamin D, especially if steroids.
	- Screen and treat other causes of osteoporosis (hyperthyroidism, hyperparathyroidism, hypogonadism)
	- Dual photon densitometry (DEXA): if possible before and at least 1, 5 and 10 year after HSCT.
	- Biphosphonate therapy if osteopenia or osteoporoses are established and if steroid therapy >7.5 mg/day is prescribed more than 3 months

## Abbreviations

GH: Growth hormone
GnRH: Gonadotrophin releasing hormone
GVHD: Graft versus host disease
HSCT: Hematopoïetic stem cell transplantation
MNGIE: Mitochondrial neurogastrointestinal encephalomyopathy
TBI: Total body irradiation
TSH: Thyroid-stimulating hormone
